# Surface Roughness and Translucency of Various Translucent Zirconia Ceramics after Hydrothermal Aging

**DOI:** 10.1055/s-0041-1736415

**Published:** 2021-12-10

**Authors:** Chaimongkon Peampring, Santiphab Kengtanyakich

**Affiliations:** 1 Department of Prosthetic Dentistry, Faculty of Dentistry, Prince of Songkla University, Hat Yai, Songkhla, Thailand

**Keywords:** zirconia, hydrothermal aging, surface roughness, translucency parameter, contrast ratio

## Abstract

**Objective** This study investigated the effect of hydrothermal aging on surface roughness and translucency of various translucent zirconia materials.

**Materials and Methods** Four types of zirconia were tested. Group 1 was translucent zirconia with no cubic structure. Group 2, 3, and 4 included cubic-containing zirconia with different amounts of cubic structures (less than 30%, 30–50%, and more than 50%, respectively). Each group contained 15 disk-shape specimens with dimensions of 15 mm in diameter and 1 mm in thickness. As-sintered surface roughness, translucency parameter, and contrast ratio were evaluated in the two different sessions, before and after aging.

**Statistical Analysis** Two-way repeated measures ANOVA with Bonferroni test was used to analyze statistically significant difference in those tested parameters. Phase structure before and after aging was analyzed by X-ray diffraction analysis (XRD).

**Results** Groups 1 and 2 showed significant increased surface roughness after aging while groups 3 and 4 showed no alteration of surface. There was no effect of aging on translucency in all groups. After aging, group 1 and 2 presented monoclinic structure (16.63 and 5.01%, respectively).

**Conclusion** Hydrothermal aging caused phase transformation and increasing surface roughness in group 1 and 2 but did not affect translucency in all groups.

## Introduction

Yttrium oxide partially stabilized zirconia (3Y-TZP) or conventional zirconia consists of 3% mol of yttrium oxide (Y _2_ O _3_ ) as a stabilizer which helps in maintaining tetragonal crystal structure at room temperature. Tetragonal zirconia has high strength and fracture toughness due to tetragonal to monoclinic transformation around the crack tip after exposed to applied stress causing crack propagation termination. 1 2 However, tetragonal zirconia has been susceptible to low temperature degradation or hydrothermal aging as it is immersed in water such as saliva. The aging process is a result of spontaneous transformation of the metastable tetragonal phase to the monoclinic in the presence of water at low temperatures which are approximately 30°C up to 400°C. 2 3 The progression of hydrothermal aging is relatively a slow process that starts on zirconia surface causing microcracks formation in the area of transformation. Crack propagation proceeds because of water penetration into the cracks leading to the intermolecular bond rupture of at the crack tip. This process results in a remarkable zirconia surface alteration and decrease in strength. 4 5 6 The effect of aging occurs obviously in the case of lower yttrium oxide content. The initial powder consisting of increased amount of yttrium oxide can create the cubic crystal formation. Since cubic zirconia is a stable phase, it could resist to hydrothermal aging and has lower ability of transformation. 7

The increased patients' aesthetic demands are driving the development of dental restorative materials from conventional zirconia which is apparently opaque to a highly translucent zirconia. Translucency is considered as an important factor in controlling aesthetics and plays an important role in decision of material selection. 8 3Y-TZP becomes more translucent by reducing size and repositioning of alumina grains resulting in improved light transmission. 7 However, its aesthetic appearance is still far from mimicking natural dentition. Another way to improve zirconia translucency is increasing the amount of Y _2_ O _3_ stabilizer that will alter the phase structure from tetragonal to cubic phase. The amount of Y _2_ O _3_ stabilizer seems to have an effect on the overall microstructure of zirconia materials. The common amounts of Y _2_ O _3_ added to improve translucency are 4 and 5% mol (4Y-TZP and 5Y-TZP). 9 Cubic crystal is the most stable phase structure; therefore, mechanical properties of cubic-containing zirconia seem to be stable after being hydrothermally aged. 10 Even though cubic-containing zirconia (4Y-TZP and 5Y-TZP) is proved to resist to hydrothermal aging, some tetragonal crystals presenting in 4Y-TZP and 5Y-TZP are prone to be transformed to monoclinic phase spontaneously after being immersed in water in long-term period especially at the zirconia surfaces. Also, there is little evidence showing that the translucency of cubic-containing zirconia is stable after aging. Effect of hydrothermal aging usually appears at the zirconia surface so the surface topography and surface roughness of zirconia with different numbers of cubic crystals could have changed after being exposed to hydrothermal aging.

Therefore, the aim of this study was to investigate the effect of hydrothermal aging on translucency parameter (TP), contrast ratio (CR), and surface roughness of various types of translucent zirconia including noncubic and cubic-containing materials. The first null hypothesis was that there was no significant effect of hydrothermal aging on surface roughness and surface topography of all zirconia groups. The second hypothesis was that hydrothermal aging did not significantly affect translucency of all zirconia groups.

## Material and Methods

### Specimen Preparation

Four different types of zirconia were tested in this study ( Table 1 ). Fifteen disk-shaped specimens were prepared from each zirconia in a partially sintered stage using a precision saw (ISOMET 4000, Buehler Ltd., Lake Bluff, Illinois, United States). All specimens were sintering completely using high temperature furnace (Zyrcomat 6000 MS, VITA Zahnfabrik H. Rauter GmbH, Bad Sackingen, Germany) according to manufacturer's instructions. The final dimensions for each test specimen were 15 mm in diameter and 1 mm in thickness confirmed with a digital calliper (ABSOLUTE Digimatic Caliper, Mitutoyo Manufacturing Company Ltd, Kawasaki, Japan). Afterward, all specimens were cleaned with ultrasonic cleaner (Model: 460/M, Elma Schmidbauer GmbH, Singen, Germany) for 10 minutes before testing. Hydrothermal aging simulation was set up using an autoclave oven (TOMY ES 215/ES-315, TOMY Kogyo) at 122°C under pressure of 2 atm for 8 hours. 10 Each specimen was sealed in a sterilization pack and placed in the autoclave. After aging, all specimens were air-dried for 24 hours before testing.

**Table 1:** Various types of zirconia and their designated group used in the present study

Group	Material	Amount of cubic (%vol)	Lot number	Manufacturers
1	3Y-TZP	0	73830	VITA Zahnfabrik. Bad Säckingen, Germany
2	4Y-TZP	<30	65890	VITA Zahnfabrik. Bad Säckingen, Germany
3	5Y-TZP	30–50	61960	VITA Zahnfabrik. Bad Säckingen, Germany
4	5Y-TZP	>50	ZB6124B	Zirkonzahn GmbH, Bruneck, Italy

### Surface Roughness Testing

Surface topography evaluation and surface roughness measurements were performed in as-sintered specimens ( *n* = 15/each group) using a noncontact optical 3D surface measurement system (Infinite Focus SL, Alicona Imaging GmbH, Graz, Austria). The measurements were performed in both before and after aging condition.

### Translucency Measurement

Translucency evaluation was interpreted from the measurement of TP and CR. TP was calculated by testing the color differences between the same specimens over the black and white backgrounds, according to the following formula 11 :

TP = [( *L* * _*B*_ − *L* * _*W*_ ) ^2^ + ( *a* * _*B*_ − *a* * _*W*_ ) ^2^ + ( *b* * _*B*_ − *b* * _*W*_ ) ^2^ ] ^1/2^

where the subscripts *B* refers to the color coordinates over the black background and *W* is the color coordinates over the white background. A higher TP value indicates a higher translucency.

The CR was calculated from the spectral reflectance of light of the specimen ( *Y* ) over black ( *Y _B_* ) and white ( *Y _W_* ) backgrounds, using the following equations:

*Y* = [( *L* * + 16)/116] ^3^ × 100

CR = *Y _B_* / *Y _W_*

The CR is 0.0 for a transparent material and 1.0 for a totally opaque material. 11

Translucency evaluation was done in the same specimens used for surface evaluation. A spectrophotometer (ColorQuest XE, Hunter Associates Laboratory, Inc., Reston, Virginia, United States) with a calibration plate and port size of 0.375 inches was used to record the CIELAB coordinates ( *L* *, *a* *, and *b* *) of zirconia disks. The TP and CR measurements were performed in both before and after aging.

### Phase Structure Analysis

Phase identifications of each group before and after aging were done by X-ray diffraction (Philips X'Pert MPD, Philips, Eindhoven, Netherlands) using Cu-Kα radiation from 0 to 90 degrees (2 *θ* ). Scans were performed at 40 kV, 30 mA, step size of 0.05 degrees/step, and a scan time of 1 s/step. The phase structures were refined as: tetragonal zirconia (t), monoclinic zirconia (m), cubic zirconia (c) and data was indexed as a library data. The crystalline phase fraction was reported as the peak position of each crystal structure. X-ray diffraction analysis (XRD) outputs were mapped with library data to detect the crystal structure on the surface of materials and volume fraction of monoclinic transformation was calculated according to studies by Toraya and Yoshimura 12 and Garvie and Nichoson. 13

### Statistical Analysis

Two-way repeated measures ANOVA with Bonferroni test was used to determine the differences in TP, CR, and surface roughness comparing before and after aging among various types of zirconia. The statistical analysis was performed using SPSS software, version 24.0 (SPSS, IBM) to detect statistically significant differences ( *α* = 0.05).

## Results

**Fig. 1:**
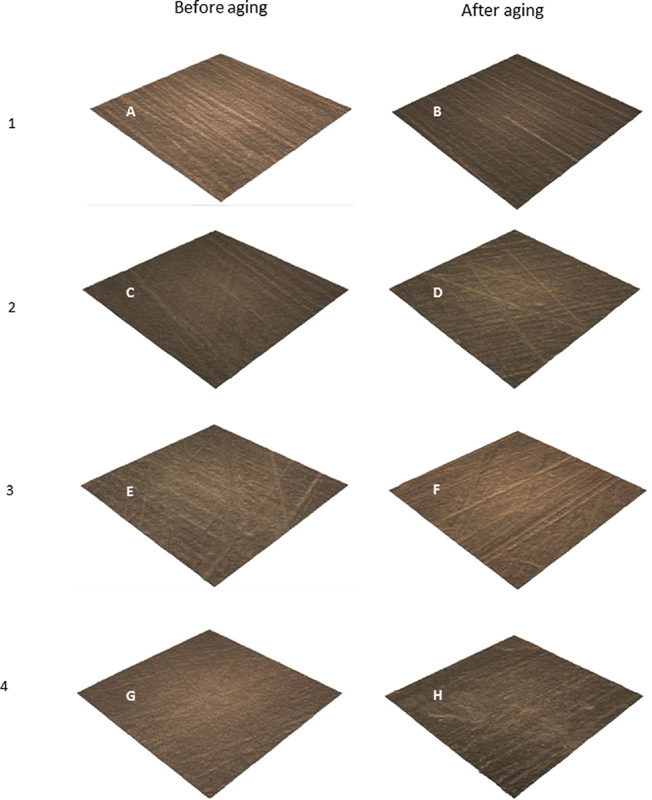
Surface topography of all experimental groups in each aging condition (surface area of 1.25 mm for testing at magnification of 50x). ( **A** ) Group 1 before aging; ( **B** ) group 1 after aging; ( **C** ) group 2 before aging; ( **D** ) group 2 after aging; ( **E** ) group 3 before aging; ( **F** ) group 3 after aging; ( **G** ) group 4 before aging; ( **H** ) group 4 after aging.

**Fig. 2:**
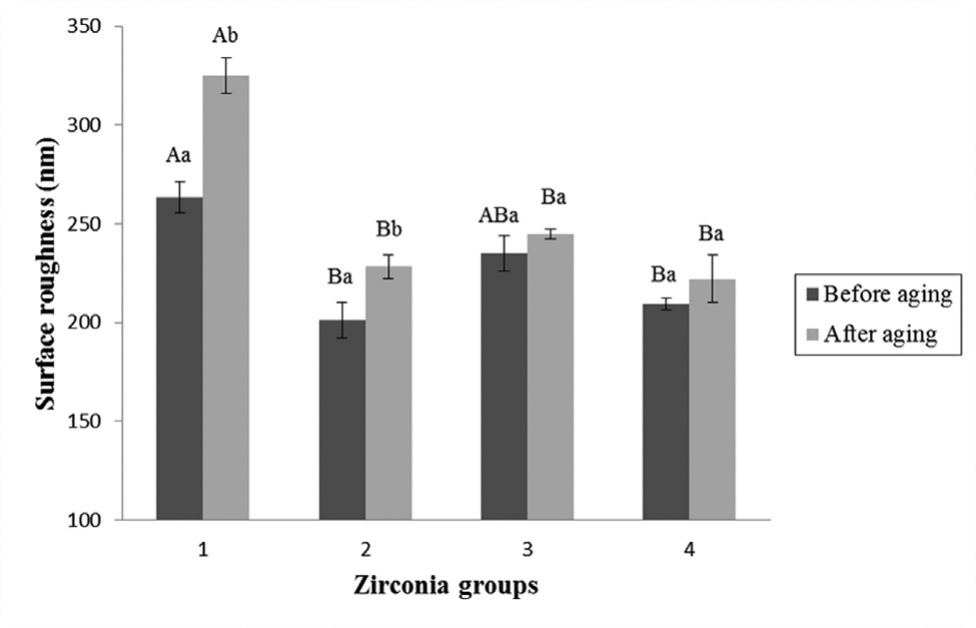
Surface roughness (Ra) values (mean ± SD) of the different experimental groups. Different uppercase letters indicate statistically significant among material group ( *p* < 0.05). Different lowercase letters indicate statistically significant comparing before and after aging within group ( *p* <0.05).

Surface topography results, which are based on visual inspection showed that group 1 presented irregular surface in all areas of testing ( Fig. 1A, B ) and followed by group 2 ( Fig. 1C, D ). However, each group revealed no changing on the surface after aging. Two-way repeated ANOVA results showed significant differences in surface roughness (Ra) values among the material groups and aging conditions ( *p* < 0.05), while there was no statistically significant interaction between two independent factors ( *p* = 0.066). Group 1 had a significant difference in surface roughness comparing between specimens in before aging condition (263.201 ± 15.888 nm) and after aging condition (325.048 ± 18.047 nm). In addition, group 2 showed significant increase in surface roughness after aging (from 201.209 ± 17.681 to 228.330 ± 12.248 nm). Groups 3 and 4 presented no alteration on surface roughness ( Fig. 2 ).

According to translucency evaluation, there was a significant difference in TP values ( Table 2 ) based on material difference ( *p* < 0.05). No significant difference based on aging conditions ( *p* = 0.156) and interaction effect ( *p* = 0.274) was detected. Group 1 showed the lowest TP value whether it was aged or not, significantly. Group 2 showed significantly lower TP value compared with groups 3 and 4 only in aged specimens. Groups 3 and 4 showed no significant difference in TP values regardless of aging condition ( *p* > 0.05). The CR values are reported in Table 3 . The results showed statistically significant difference between the material groups ( *p* < 0.05). The mean CR value of group 1 was significantly the highest value. The CR values can be ranked as follow: group 4 = 3 <2 <1 regardless of aging condition. After aging, CR values appeared to be no change statistically comparing within material group ( *p* = 0.126). Therefore, there was no significant change in translucency within material group comparing before and after aging.

**Table 2:** Mean ± SD of translucency parameter (TP) in all groups

Group	Translucency parameter	
	Before aging ( *n* = 15)	After aging ( *n* = 15)
1	6.574 ± 0.867 ^Aa^	6.268 ± 0.821 ^Aa^
2	9.794 ± 0.226 ^Ba^	9.649 ± 0.216 ^Ba^
3	10.001 ± 0.319 ^Ba^	10.068 ± 0.289 ^Ca^
4	10.018 ± 0.772 ^Ba^	10.099 ± 0.253 ^Ca^

Note: Different uppercase letters in the same column indicate statistically significant difference ( *p* < 0.05); Different lowercase letters in the same row indicate statistically significant difference ( *p* < 0.05).

**Table 3:** Mean ± SD of contrast ratio (CR) in all groups

Group	Contrast ratio	
	Before aging ( *n* = 15)	After aging ( *n* = 15)
1	0.838 ± 0.021 ^Aa^	0.847 ± 0.020 ^Aa^
2	0.745 ± 0.005 ^Ba^	0.746 ± 0.005 ^Ba^
3	0.728 ± 0.007 ^Ca^	0.729 ± 0.006 ^Ca^
4	0.726 ± 0.008 ^Ca^	0.727 ± 0.020 ^Ca^

Note: Different uppercase letters in the same column indicate statistically significant difference ( *p* < 0.05); Different lowercase letters in the same row indicate statistically significant difference ( *p* < 0.05).

**Fig. 3:**
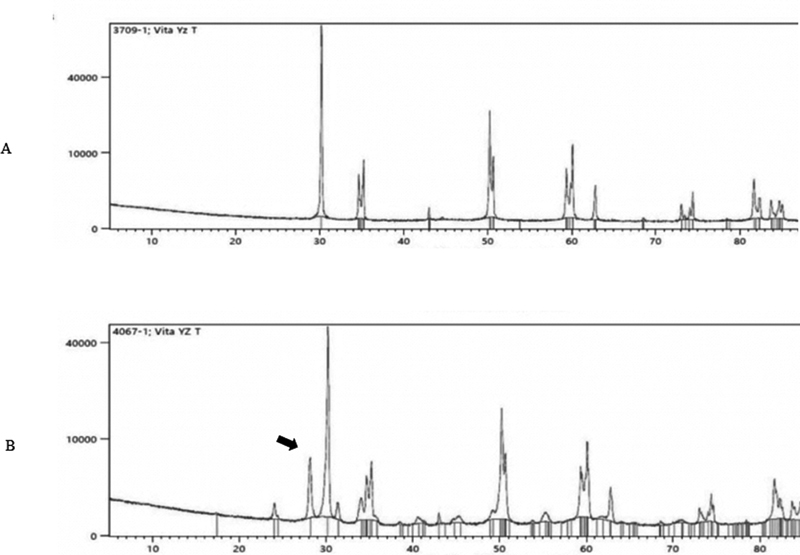
Phase structure from XRD results for group 1. The black arrow indicates peak of monoclinic phase, ( **A** ) before aging, ( **B** ) after aging. XRD, X-ray diffraction.

**Fig. 4:**
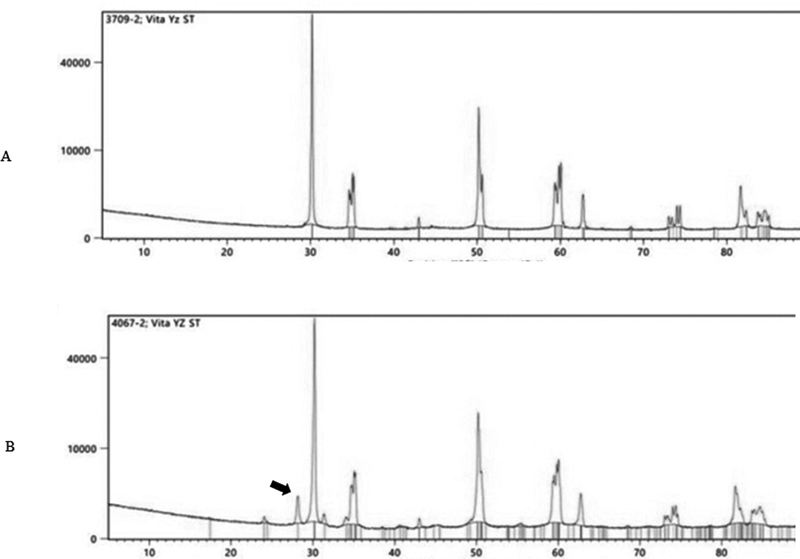
Phase structure from XRD results for group 2. The black arrow indicates peak of monoclinic phase, ( **A** ) before aging; ( **B** ) after aging. XRD, X-ray diffraction.

The phase structure analysis using XRD showed that no monoclinic phase was detected in all groups before aging. After aging, the peaks representing monoclinic structure were seen in groups 1 and 2 ( Figs. 3 and 4 ). The amount of monoclinic phase detected in groups 1 and 2 were up to 16.63 and 5.01%, respectively. There was no detected monoclinic phase after aging in groups 3 and 4.

## Discussion

Conventional zirconia containing 3% mol of yttrium oxide retains tetragonal crystal and no cubic crystal presented at room temperature. A benefit of having tetragonal crystal structure is the transformation toughening of zirconia material. 14 15 Therefore, 3Y-TZP has superior mechanical properties compared with other dental ceramics. 16 17 18 A drawback of retaining tetragonal phase structure at room temperature is opacity of a material thus it can be used as a high strength substructure of a crown which has to be veneered with higher aesthetic materials. 19 20 Zirconia translucency can be improved by either modifying microstructure or increasing the amount of Y _2_ O _3_ . 21 Translucent zirconia tested in this study could be varied based upon the amount of cubic structure. Group 1 was zirconia with no cubic crystal while group 2, 3, and 4 included cubic-containing zirconia with different amount of cubic crystal (30%, 30–50%, and more than 50%, respectively). Since, hydrothermal degradation, a spontaneous transformation from the metastable tetragonal to the monoclinic structured facilitated by oral fluid, usually takes place on the zirconia surface, this study aimed to evaluate surface alteration and translucency change of all experimental group under extreme *in vitro* aging conditions. This accelerated hydrothermal aging was performed in an autoclave at 122°C under constant pressure of 2 atm for 8 hours. It is possible to assume that 1 hour of autoclaving has the same effect as 1 year in clinical services. 6

Surface roughness evaluation among various types of translucent zirconia and aging condition showed significant difference; therefore, the first null hypothesis was rejected. To eliminate the influence of surface polishing protocol, the surface roughness in this study had been tested on as-sintered specimens. The results showed that hydrothermal aging affected the Ra in group 1 and 2 due to the progressive spontaneous transformation of the metastable tetragonal phase to the monoclinic phase in the presence of oral fluid. 2 22 This could be confirmed by XRD results showing detected monoclinic in group 1 and 2 after aging. It is in accordance with some previous studies showing that surface deterioration occurred in a zirconia material. 23 Groups 3 and 4 retained large amounts of cubic structure, the most stable crystal structure; at room temperature thus phase transformation would not occur after being accelerated by hydrothermal aging. A threshold surface roughness below 0.2 µm could be considered as a smooth surface, in which, no further bacterial accumulation could be expected. 24 25 The roughness threshold detectable by the tongue was more than 0.5 µm. 26 Mean Ra values of aged specimens in group 1 (0.325 µm) and 2 (0.228 µm) were less than the threshold that can be detected by tongue.

The second null hypothesis was accepted because the optical properties of the cubic zirconia material were not influenced by hydrothermal aging. There was no significant effect of aging on translucency of zirconia. Translucency of restorative material affects esthetic outcome of restorations by mimicking natural appearance. Patients are concerned more about esthetic outcome so translucency of zirconia material should be investigated. 27 Residual porosity and zirconia grain size are the main factors controlling light transmission. Transformation of tetragonal crystal to monoclinic crystal results in increasing grain size. 22 Therefore, the assumption of hydrothermal aging affecting zirconia translucency could be possible. However, the transformation occurred only at the surface of material which was not affecting the overall grain size and translucency which was confirmed by this study. Zirconia translucency was evaluated by measuring TP and CR. The TP was determined by calculating the color differences in the same specimen against black and white backgrounds. A higher TP value indicated a higher translucency. On the contrary, the CR was calculated from the light spectral reflectance of the same specimen over a black and a white background, the CR is range from 0.0 and 1.0, in which 0.0 means a material is transparent material and 1.0 means a material is totally opaque. 11 Group 1 presented less translucent than cubic-containing groups (2, 3, and 4) and group 2 presented no significant difference in TP values compared with groups 3 and 4. However, the CR in group 2 was lower than groups 3 and 4. These results could be explained in which TP value was determined based on CIELAB color parameter using *L* * *a* * *b* *data. Nevertheless, *a* * and *b* * are hue values which were achieved from color pigments. Each material contained different amount of color pigments resulting in a different hue value. This might affect the TP value. This study showed that zirconia with cubic structure had high translucency since the cubic zirconia is optically isotropic without light scattering at the grain boundaries. 21 23 Thus, zirconia ceramics with a slight variation in cubic structure resulted in a substantially different translucency. The strategy of increasing the amount of cubic crystals resulted in higher translucency of zirconia. 28 29 A previous study concluded that high translucency monolithic zirconium oxide materials had TP values in the range of 14.6 to 23.2. 30 TPs of our study showed lower values than the previously mentioned study because of different surface finishing protocols resulting in differences in smoothness of the surfaces, leading to decreased light scattering, and higher TP values. 31 All specimens in this study were tested in as-sintered condition; therefore, this could be a cause of lower translucency.

Phase transformation after aging was confirmed with X-ray diffraction analysis. The results showed that there was no monoclinic phase presented after hydrothermal aging in groups 3 and 4. The results indicated that the high amount of cubic crystal caused a more stable condition of zirconia; therefore, phase transformation did not occur. The present results were in accordance with a previous study evaluating the aging stability of 5Y-TZP. The authors reported that cubic-containing zirconia did not show any transformation after 300 hours of accelerated aging. 6 In our study, group 2 exhibited some monoclinic crystals (approximately 5.01%) after hydrothermal aging for 8 hours, which was possible because of less amount of yttrium oxide stabilizer added. 16 For group 1, the presence of monoclinic phase after autoclave aging coincided with the previous study which evaluated the amount of monoclinic phase on XRD after 8 hours of aging. 10 They also found that hydrothermal aging did not alter mechanical properties of cubic-containing zirconia. 10 However, this study found that the hydrothermal aging resulted in surface alteration of noncubic translucent zirconia and cubic-containing zirconia which consisted of less than 30% of cubic crystals. Further study should investigate the effect of hydrothermal aging on surface gloss of highly polished zirconia.

## Conclusion

Within the limitations of this *in vitro* study, the following conclusions were drawn: hydrothermal aging in autoclave for 8 hours caused phase transformation and increasing surface roughness only in group 1 (noncubic) and 2 (cubic less than 30%). However, surface roughness of groups 3 and 4 was not affected by hydrothermal aging. Also, hydrothermal aging did not affect translucency of all zirconia materials.
